# Reasons for tooth extraction in a Swedish county dental service: a 5-year longitudinal cohort study with focus on endodontic pathology

**DOI:** 10.1038/s41405-026-00430-3

**Published:** 2026-04-25

**Authors:** Simon Persson, Fernando Mota De Almeida, Pontus Lundqvist, Anna Levinsson, Emine Camci, EndoReCo, Thomas Kvist, Emma Wigsten

**Affiliations:** 1 Public Dental Service, Västra Götaland Region, Gothenburg, Sweden; 2 Tandvårdens Kompetenscentrum, Norrbotten, Public Dental Service, Luleå, Sweden; 3 Department of Endodontology, Institute of Odontology, The Sahlgrenska Academy, University of Gothenburg, Gothenburg, Sweden; 4 Division of Pediatric Dentistry, Department of Dental Medicine, Karolinska Institutet, Huddinge, Sweden; 5 Department of Epidemiology, Biostatistics and Occupational Health, School of Population and Global Health, Faculty of Medicine and Health Sciences, McGill University, Montreal, QC Canada

**Keywords:** Root canal treatment, Dentoalveolar surgery

## Abstract

**Aims:**

The aim of this study was to investigate tooth extractions in a Swedish public general dental practice setting, including the proportion of endodontically treated teeth, reasons for extraction, and subsequent prosthetic replacement.

**Materials and methods:**

A prospective cohort study was conducted in 20 clinics within the Public Dental Service of Västra Götaland, Sweden. During an 8-week period, general dental practitioners consecutively registered reasons for tooth extraction. Patients’ pain levels were assessed. Pre-extraction radiographs were assessed for tooth status. Five-year follow-up data from electronic dental records were used to determine whether the extracted teeth had been prosthetically replaced and to classify the type of replacement. Descriptive and inferential statistics were used.

**Results:**

A total of 133 patients (61 men and 72 women; mean age 54.0 years, SD = ± 15.8) underwent extractions. Endodontic pathology (36.8%) and fractures (24.8%) were the most common reasons. Sixty-one patients had previous endodontic treatment, and one-third of extracted teeth were root-filled. Thirty-five teeth were prosthetically replaced, most often with removable prostheses (45.7%).

**Conclusions:**

Endodontically treated teeth, including those with initiated or completed root canal treatment, were markedly overrepresented among extractions, yet prosthetic replacement was infrequent. Younger patients less often opted for replacement, warranting further investigation of factors influencing replacement decisions.

## Key Points

- Endodontically treated teeth were markedly overrepresented among extractions in a public dental practice setting
- Prosthetic replacement was infrequent, mainly with removable prostheses
- Younger patients less often chose prosthetic replacement post-extraction

## Introduction

Oral health has improved significantly in Sweden over recent decades [1, 2]; nevertheless, tooth extraction remains a common procedure in general dental practice. In 2024, 642,196 tooth extractions were performed among adults, according to data from the Swedish Social Insurance Agency [3]. Although the reasons for tooth extraction have been investigated in various populations [4, 5], there is a knowledge gap of data concerning extractions performed within general dental practice.

Root-filled teeth are at greater risk of extraction compared to their non-root-filled counterparts [6, 7]. Longitudinal Scandinavian studies have demonstrated that fewer than 1% of non-root-filled are extracted during 6 years of follow-up, whereas 7% of root-filled teeth were lost during the same interval, and 14% after 10 years of follow-up [8, 9]. The indications for extraction vary, but crown or root fractures and persistent endodontic pathology are most frequent [10–13].

More recently, several large-scale register-based studies, including three from Sweden, have reported that root-filled teeth exhibit an annual extraction rate of approximately 2% in the adult population [14–20]. Further, root canal treatment remains a challenging procedure within general dental practice, particularly for molar teeth, as many are extracted before completion of the root-filling procedure [12, 21–23]. A recent report from a Swedish Public Dental Service organisation found that 13% of teeth undergoing root canal treatment were extracted rather than completed within one year of initiation [12].

This highlights the need to assess the proportion of extracted teeth that have undergone endodontic treatment, including both initiated and completed cases. In addition, the main reasons for extraction warrant further investigation. Consideration of these aspects is vital when evaluating treatment outcomes, risks, and the costs associated with root canal procedures in general dental practice.

Finally, some extracted teeth may ultimately be replaced with prosthetic replacements. Decisions regarding tooth replacement are influenced by several factors, including the number of remaining teeth and the anatomical position of the extracted tooth [4, 24]. Nevertheless, there is a lack of studies investigating which extracted teeth that are subsequently replaced, particularly within the context of public general dental care.

The aim of this study was to investigate tooth extractions in a Swedish general dental practice setting, with a focus on endodontic status and subsequent prosthetic replacement. The specific objectives were to (1) determine the proportion of extracted teeth with previous endodontic treatment, (2) describe the reasons for extraction in relation to endodontic status, and (3) evaluate whether extracted teeth were prosthetically replaced within five years, and if so, by which modality.

## Material and methods

The study was designed as a prospective cohort study, with baseline defined at the time of tooth extraction and a register-based follow-up period of five years. The study was conducted in accordance with the STROBE statement and guidelines [25].

### Ethical considerations

The study protocol was approved by the Regional Ethical committee in Gothenburg, Sweden, in 2015 (Dnr: 817-16). Written informed consent was obtained from all participants prior to inclusion. No monetary incentives were provided for participation. The authors declare no conflicts of interest.

### Participating general dental care clinics

Twenty clinics within the Public Dental Service of Västra Götaland Region, Sweden, participated, representing 21% of the region’s public clinics (*n* = 109, in 2015). The clinics covered a broad geographical and socioeconomic range. Five were in Gothenburg (population ~570,000), while the remaining fifteen were situated in smaller towns and rural areas across Västra Götaland. A total of 113 general dental practitioners (GDPs) were involved in recruiting patients for the study.

### Participant recruitment

Participants were consecutively enrolled when undergoing tooth extraction during the designated recruitment period. Each clinic conducted screening over an eight-week period, with start dates ranging from May 2015 to December 2016.

**Fig. 1:**
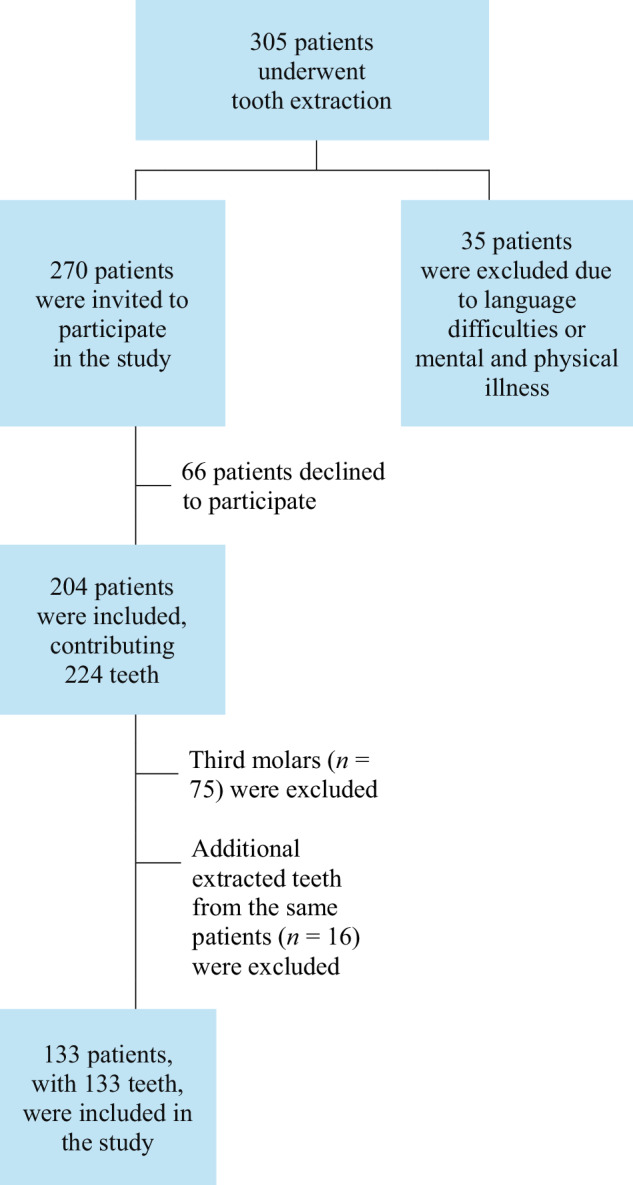
Flow chart of patient inclusion. A total of 305 patients underwent tooth extractions during the 8-week period. Of these, 270 were invited to participate; 35 were excluded due to language barriers or physical/mental illness, and 66 declined participation. Subsequently, 204 patients consented, contributing 224 teeth. Third molars (*n* = 75, from 73 patients), and additional extracted teeth (*n* = 16, from 13 patients) were excluded to ensure only one tooth per patient was analysed. The final study cohort included 133 patients, each contributing one non-third molar tooth.

Patients were eligible and invited to participate if they had a tooth extracted during the clinic’s recruitment period, were aged ≥18 years, and could provide written informed consent in Swedish. A flowchart over the recruitment process is presented in Fig. 1. In patients with multiple extracted teeth, only the first tooth treated was included.

### Data collection and patient characteristics

#### Baseline data

**Fig. 2:**
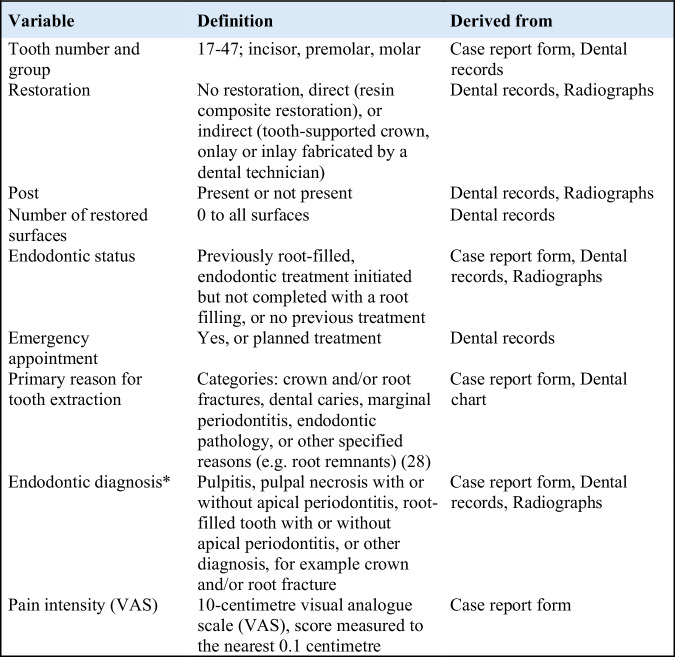
Definitions and sources of study variables. Variable definitions and sources; *The conditions were designated as either symptomatic or asymptomatic based on endodontic diagnosis, reported pain intensity (VAS) or dental records.

For each included patient, the GDP recorded the type of tooth that was extracted and the reason for extraction, on a case report form, based on clinical judgement and a classification system used in Sweden (Fig. 2; [26, 27]). When multiple reasons were reported for the same tooth, the authors (SP, EW) jointly reviewed the clinical records to identify the primary reason. A hierarchical classification strategy was used, whereby conditions such as root fractures - being less amenable to treatment - were ranked above more manageable conditions like dental caries. The categorisation followed the methodology outlined by Landys Borén et al. [28].

Patient-reported pain intensity was recorded using a 10-centimetre visual analogue scale (VAS), where 0 represents ‘no pain’ and 10 represents ‘the worst possible pain’. Scores were recorded to the nearest decimal. Further variable definitions are presented in Fig. 2.

All other baseline variables were retrospectively extracted from dental records obtained from the Public Dental Service. Patient-specific variables including sex, age, the number of remaining teeth (including third molars) and whether tooth extraction was performed at an emergency or a scheduled appointment, were recorded.

#### Five-year follow-up data

Each tooth was monitored in the digital dental records of the Public Dental Service from baseline until the last available clinical entry, recorded on 20 December 2021. When the edentulous space was restored, the date and type of prosthetic replacement was documented and classified as one of the following: dental implant, fixed dental prosthesis (FDP; tooth-supported), removable dental prosthesis, orthodontic treatment for space closure, or splinted tooth. If a patient initially received a removable prosthesis that was subsequently replaced by an FDP or implant during the follow-up period, only the final replacement modality was recorded.

#### Radiographs

Available digital radiographs were reviewed to assess the presence of root filling material and/or a post at baseline, and to confirm the type of prosthetic replacement at follow-up. The software Weasis DICOM Medical Viewer version 4.5.1 was used. Evaluations were performed jointly by authors SP, EC, and EW, allowing for cross-validation. In cases of uncertainty, findings were discussed until consensus was reached.

#### Classification of groups in respect to previous endodontic treatment

Based on all recorded data, the extracted tooth was classified as either root-filled, endodontic treatment initiated, or having no previous endodontic treatment. As the study was based on questionnaires, routine clinical dental records and radiographs, no detailed assessment of the underlying causes of endodontic failure was performed.

### Statistical analysis

No formal sample size calculation was conducted. Instead, a pragmatic approach prioritised feasibility, aiming to recruit patients who met the inclusion criteria. Although recruitment occurred across multiple clinics, no formal cluster sampling was used; standard methods assuming independent observations were applied.

Unavailable data were treated as missing without imputation, and data were analysed without transformation.

Descriptive statistics were presented as count and percentage (categorical data) or mean, standard deviation (SD), median, minimum and maximum (continuous data), as appropriate. Group comparisons were conducted using independent samples t-test for continuous variables and Fisher’s exact test for categorical variables. All statistical tests were two-sided and conducted at the 5% significance level. Statistical analyses were performed using R version 4.2.1 [29].

### Ethics approval statement and document

This study was approved by the Regional Ethical Committee in Gothenburg, Sweden (Dnr: 817-16). The research has been conducted in full accordance with ethical principles, including the World Medical Association Declaration of Helsinki (version 2008) and the requirements of Swedish law, under which the research has been conducted. All participating patients have received written and oral information about the study and have provided their verbal and written informed consent. The data do not contain any information that could identify the participants.

## Results

During the predefined study period, 305 individuals underwent tooth extraction at the participating clinics. Of these, 270 were invited to participate and 204 consented, providing 224 extracted teeth. After excluding 75 third molars and selecting one tooth per individual, the final sample comprised 133 patients and 133 teeth, of which 61 men and 72 women, with a mean age of 54.0 years (Fig. 1; Table 1). Among the included patients, 110 (82.7%) attended scheduled recall appointments, whereas 23 (17.3%) sought care only on an emergency basis. Most extractions were performed during scheduled appointments (*n* = 92, 69.2%), although 47 patients (35.3%) had attended an emergency appointment prior to extraction. Pain was reported by 59 patients (44.4%), with a mean intensity of 3.9 ± 2.5 at the time of extraction. Most extracted teeth were molars (*n* = 80, 60.2%), and had a direct restoration (*n* = 68, 51.1%). Endodontic pathology was the most common reason for extraction in 49 cases (36.8%).

**Table 1:** Patient- and tooth-based characteristics at the time of tooth extraction, according to endodontic status

	Total	Endodontic treatment group^a^			*p*-value
		Root-filled	Treatment initiated	No endodontic treatment	
**Variable**	*n* = 133	*n* = 45 (34.1%)	*n* = 16 (12.1%)	*n* = 71 (53.8%)	
**Patient-based characteristics**					
Sex, *n* (%)					0.705
Male	61 (40.7%)	20 (44.4%)	9 (56.2%)	32 (45.1%)	
Female	72 (59.3%)	25 (55.6%)	7 (43.8%)	39 (54.9%)	
Age (years)					0.008
mean, (SD)	54.0 (15.8)	59.0 (13.5)	45.5 (17.3)	53.0 (16.0)	
median (min;max)	55.0 (19;84)	59.0 (23;84)	46.0 (20;79)	56.0 (19;83)	
Age category, *n* (%)					0.023
Below 40 year*s*	27 (20.3%)	3 (6.7%)	6 (37.5%)	17 (23.9%)	
40–59 years	52 (39.1%)	20 (44.4%)	7 (43.8%)	25 (35.2%)	
60 years or older	54 (40.6%)	22 (48.9%)	3 (18.8%)	29 (40.8%)	
Number of remaining teeth					0.115
mean, (SD)	23.7 (4.4)	24.2 (3.3)	25.2 (3.5)	23.0 (5.1)	
median (min;max)	25.0 (3;28)	25 (14;28)	26 (13;28)	25 (3;27)	
Emergency appointment, *n* (%)					0.687
Yes	41 (30.8%)	12 (26.7%)	4 (25%)	24 (33.8%)	
Presence of diagnosed symptoms, *n* (%)					0.036
Asymptomatic	61 (45.9%)	29 (64.4%)	5 (31.2%)	27 (38%)	
Symptomatic	59 (44.4%)	15 (33.3%)	10 (62.5%)	33 (46.5%)	
Missing, *n* (%)	13 (9.8%)	1 (2.2%)	1 (6.2%)	11 (15.5%)	
Pain intensity (VAS)					
Symptomatic/mean (SD)	3.9 (2.5)	2.5 (2.3)	4.8 (2.2)	4.3 (2.4)	0.027
median (min;max)	4.0 (0.5;9.5)	1.5 (0.5;8.0)	5.5 (1.0;7.0)	4.5 (1.0;9.5)	
Missing, *n* (%)	6 (4.5%)	1 (2.2%)	1 (6.3%)	4 (5.6%)	
**Tooth-based characteristics**					
Primary reasons for tooth extraction based on dental records, *n* (%)					<0.001
Crown and/or root fractures	33 (24.8%)	19 (42.2%)	7 (43.8%)	7 (9.9%)	
Dental caries	15 (11.3%)	1 (2.2%)	1 (6.2%)	13 (18.3%)	
Periodontal pathology	9 (6.8%)	1 (2.2%)	0 (0%)	8 (11.3%)	
Endodontic pathology	49 (36.8%)	12 (26.7%)	7 (43.8%)	29 (40.8%)	
Other^b^	25 (18.8%)	12 (26.7%)	0 (0%)	13 (18.3%)	
Missing, *n* (%)	2 (1.5%)	0 (0%)	1 (6.2%)	1 (1.4%)	
Jaw, *n* (%)					0.611
Maxilla	78 (58.6%)	24 (53.3%)	10 (62.5%)	44 (62%)	
Mandible	55 (41.4%)	21 (46.7%)	6 (37.5%)	27 (38%)	
Tooth group, *n* (%)					0.197
Incisor/canine	16 (12%)	3 (6.7%)	0 (0%)	13 (18.3%)	
Premolar	37 (27.8%)	14 (31.1%)	4 (25%)	19 (26.8%)	
Molar	80 (60.2%)	28 (62.2%)	12 (75%)	39 (54.9%)	
Previous restoration, *n* (%)					<0.001
No restoration	37 (27.8%)	5 (11.1%)	2 (12.5%)	30 (42.3%)	
Direct restoration	68 (51.1%)	23 (51.1%)	13 (81.2%)	31 (43.7%)	
Indirect restoration	28 (21.1%)	17 (37.8%)	1 (6.2%)	10 (14.1%)	
Number of restored surfaces, *n* (%)					<0.001
None	37 (27.8%)	5 (11.1%)	2 (12.5%)	30 (42.3%)	
1–2	30 (22.6%)	6 (13.3%)	5 (31.2%)	18 (25.4%)	
3–4	27 (20.3%)	8 (17.8%)	8 (50%)	11 (15.5%)	
All surfaces	39 (29.3%)	26 (57.8%)	1 (6.2%)	12 (16.9%)	

*max* maximum, *min* minimum, *SD* standard deviation, *VAS* visual analogue scale.

^a^Endodontic treatment history was based on case report form, dental records and radiographs. Missing data regarding endodontic status was limited to one individual.

^b^Other reasons for extraction included: root remnants (*n* = 16), fractured direct restorations (*n* = 3), orthodontic treatment (*n* = 3), extensive loss of tooth-substance (*n* = 2), and food-impaction (*n* = 1); Fisher’s exact test was used for categorical variables.

### Distribution of extracted teeth across prior endodontic treatment

Endodontic status was missing for one tooth (0.8%) in the group comparison analyses (*n* = 132). Sex, number of remaining teeth, emergency versus planned appointments, jaw, and tooth group did not differ significantly across the categories root-filled, endodontic treatment initiated, or no previous endodontic treatment. All other patient- and tooth-based variables showed statistically significant variation across these categories (*p* < 0.05; Table 1). The highest mean age occurred in the root-filled group (59.0 ± 13.5 years), whereas the lowest was in the endodontic treatment-initiated group (45.5 ± 17.3 years).

Proportion of symptomatic teeth and mean pain intensity were highest in the endodontic treatment-initiated group (62.5%; 4.8 ± 2.2) and lowest in the root-filled group (33.3%; 2.5 ± 2.3).

Molars were the most prevalent teeth across all three groups, with the highest representation observed in the endodontic treatment-initiated group (*n* = 12, 75.0%). Similarly, directly restored teeth were common in all groups, but were most frequently observed in the endodontic treatment-initiated group (*n* = 13, 81.2%).

Crown and root fractures were the most common reasons for extraction in both root-filled teeth (*n* = 19, 42.2%) and in the endodontic treatment-initiated group (*n* = 7, 43.8%). Among teeth without signs of endodontic treatment, endodontic pathology predominated (*n* = 29, 40.8%), and it was likewise common in the endodontic treatment-initiated group (*n* = 7, 43.8%). In contrast, only 12 root-filled teeth (26.7%) were extracted due to endodontic pathology.

### Prosthetic replacement after tooth extraction

Within five years of extraction, 35 teeth (26.3%) were prosthetically replaced. No significant differences were found between men and women, or between age groups (Table 2). Anterior teeth were most frequently replaced (*n* = 14, 87.5%), followed by premolars (*n* = 13, 35.1%), while molar replacement was less common (*n* = 8, 10.0%; *p* < 0.001).

**Table 2:** Patient- and tooth-based characteristics at the time of tooth extraction, according to prosthetic replacement

Variable	Prosthetic replaced	No replacement	*p*-value
	*n* = 35 (26.3%)	*n* = 98 (73.7%)	
**Patient-based characteristics**			
Sex, *n* (%)			0.313
Male	13 (21.3%)	48 (78.7%)	
Female	22 (30.6%)	50 (69.4%)	
Age category, *n* (%)			0.482
Below 40 years	7 (25.9%)	20 (74.1%)	
40–59 years	11 (21.2%)	41 (78.8%)	
60 years or older	17 (31.5%)	37 (68.5%)	
**Tooth-based characteristics**			
Endodontic treatment group^a^			0.398
Previous root-filled	13 (28.9%)	32 (71.1%)	
Treatment initiated	2 (12.5%)	14 (87.5%)	
No endodontic treatment	20 (28.2%)	51 (71.8%)	
Tooth group, *n* (%)			<.001
Incisor/canine	14 (87.5%)	2 (12.5%)	
Premolar	13 (35.1%)	24 (64.9%)	
Molar	8 (10%)	72 (90%)	
**Tooth-specific replacement**			
Type of prosthetic replacement			
Dental implant	5 (14.3%)	-	
Tooth-supported prosthesis	10 (28.6%)	-	
Removable prosthesis	16 (45.7%)	-	
Orthodontic space closure	3 (8.6%)	-	
Splinted tooth	1 (2.9%)	-	

*max* maximum, *min* minimum, *SD* standard deviation

^a^Endodontic treatment history was based on case report form, dental records and radiographs. Missing data regarding endodontic status was limited to one individual.

No statistical test of difference in distribution between groups was carried out for the Type of prosthetic replacement due to the sparsity of data; Fisher’s exact test was used for the categorical variables.

Removable prostheses were the most common prosthetic replacement (*n* = 16, 45.7%), followed by tooth-supported fixed prostheses (*n* = 10, 28.6%) and dental implants (*n* = 5, 14.3%; Table 2).

## Discussion

In this prospective follow-up study of 204 patients undergoing tooth extractions in a public general dental practice setting, third molars accounted for 75 teeth (33.5%), making them the most frequent tooth group - reflecting particular considerations. They were excluded from the analyses, as they are infrequently root-filled or prosthetically restored [30].

Among the remaining extracted teeth, three endodontic categories became apparent: permanently root-filled teeth (*n* = 45; 34.1%), teeth with initiated endodontic treatment (*n* = 16; 12.1%), and teeth without any prior endodontic treatment (*n* = 71; 53.8%), with prior status missing for one tooth (0.8%). Endodontic pathology was the leading reason for extraction overall, accounting for over one-third of cases, while crown and root fractures represented approximately one-quarter of all extractions and were the primary reason for extraction in 4 out of 10 root-filled teeth. Molars continued to be the most frequently extracted teeth [10–12, 18, 19]. Only around one-quarter of extracted teeth were prosthetically replaced within five years, with replacements occurring predominantly in the anterior region.

Over the past decades, improvements in oral health have enabled more individuals to retain their natural teeth into older age [1, 2]. Nonetheless, the greater retention of teeth has increased the number of teeth vulnerable to dental diseases, contributing to a higher prevalence of dental conditions among the ageing population. Hence, it is unsurprising that the majority of extracted teeth in this study showed evidence of previous dental treatment, as dental caries can lead to extensive restorations that may ultimately necessitate endodontic procedures, which, although often successful [31, 32], can leave residual pathology, further compromise tooth structure, increase the risk of fractures, and render extraction the necessary or most appropriate solution. Ergo, improvements in oral health may have deferred tooth extractions to later in life.

Reasons for tooth extraction have been investigated across various populations [4, 5]. The findings are consistent with previous studies, as just under half of the extracted teeth had undergone endodontic treatment, most commonly being root-filled [8, 9, 31, 32]. Furthermore, more than one-third of the extractions were associated with endodontic pathology, including conditions such as pulpitis, pulpal necrosis, and previously root-filled teeth with or without clinical or radiographic findings of apical periodontitis. These results also align with earlier studies conducted in similar settings [10–13], the clinical significance of endodontic pathology as a major contributor to tooth loss even in a general practice setting.

Most root-filled teeth in this study were asymptomatic at the time of extraction, which is consistent with studies reporting low frequencies of persistent pain [33–35]. Root-filled teeth were primarily extracted due to crown and or root fractures and endodontic pathology, in contrast to teeth without prior endodontic treatment. This finding aligns with studies performed in general dental practice, where fractures, endodontic failure and caries have been identified as a primary reason for extraction of root-filled teeth [10–12]. Similar tendencies were observed in a Swedish population-based study where non-restorable root-filled teeth were preferentially extracted, while symptomatic salvageable teeth underwent endodontic retreatment, potentially reflecting differences in pain management during primary endodontics [36].

The higher proportion of symptomatic teeth and greater pain intensity in the endodontic treatment-initiated group may suggest persistent acute symptoms after initial emergency treatment, whereas root-filled teeth were more often extracted for structural or restorative reasons. These findings should be interpreted cautiously and do not permit causal inference.

An important factor that may contribute to the extraction of endodontically treated teeth is the quality and extent of the coronal restoration. In the present study, crown and or root fractures were a common reason for extraction among root-filled teeth. Notably, only a minority of these teeth had indirect restorations, and fewer than 60% had full-coverage restorations, suggesting that a substantial proportion may have lacked adequate cuspal coverage. It is well established that endodontically treated teeth without sufficient cuspal protection are more prone to fracture [18–20], which is a common cause of tooth loss. In addition, inadequate coronal restoration may increase the risk of leakage and reinfection, potentially compromising long-term outcomes of endodontic treatment. Although the present study was not designed to assess restoration quality in detail, these findings highlight the potential importance of appropriate post-endodontic restorative strategies.

Extraction of root-filled teeth due to endodontic pathology, despite the absence of symptoms, raises questions regarding the decision-making process [37–39]. Scandinavian cross-sectional studies have reported apical periodontitis in 25–50% of root-filled teeth across different general dental practice settings [40–43]. However, longitudinal data suggest that the frequency of non-surgical or surgical re-treatment procedures remains relatively low [20, 36, 38]. For example, a Swedish national register-based study of 215,611 individuals reported non-surgical re-treatment rates of 3.5% and surgical re-treatment rates of 1.4% over a 10–11-year follow-up period [20]. Similarly, a study conducted in the northern Sweden within the Public Dental Service reported a 6% prevalence of non-surgical re-treatment over a 10-year period [36].

Extraction due to apical periodontitis in previously endodontically treated teeth should be interpreted in light of known causes of persistent or recurrent disease. Factors such as incomplete root canal disinfection, inadequate obturation, coronal leakage, or secondary microbial contamination are commonly implicated in the persistence of apical pathology [27]. In the present study, a substantial proportion of these teeth were molars, which are known to present greater anatomical complexity and may be perceived as more challenging [23, 27]. Although the present study did not allow assessment of technical or biological factors, these mechanisms provide context for why previously treated teeth may develop persistent disease and ultimately require extraction.

In contrast to the relatively low re-treatment rates, the frequency of extractions of root-filled teeth appears to be considerably higher. Considering that root-filled teeth constitute only about a maximum of 19% of the dentition in the general population [1], their marked overrepresentation among extracted teeth should not be interpreted solely as treatment failure. Instead, this may reflect cumulative therapeutic efforts to prolong tooth survival prior to extraction, as root-filled teeth are typically extracted at older ages - consistent with extended retention in populations with good access to dental care. Swedish observational studies conducted in general dental practice report a mean annual incidence of approximatively 2% [10, 11, 18, 20, 38], with molars being of particular concern [10–12, 18, 23]. This may be attributed to several factors, including the often suboptimal technical quality of the root fillings and the absence of periapical healing in root-filled molars [7, 42, 44].

The frequency of prosthetic replacement was low in this cohort; lower than reported in a comparable Belgian population-based study [45]. Nonetheless, potential explanatory factors include the generally good oral health in the studied region, reflected by the number of remaining teeth, as well as the distribution of the extracted teeth, a majority being molars [4, 24]. This pattern may be associated with the overall dentition retained by most of patients, as a threshold of approximately 20 teeth - commonly referred to as short dental arch - is often considered sufficient to maintain functional oral health [46, 47]. Anterior teeth were most frequently replaced, typically within the first six months following extraction. This likely reflects their essential role in both aesthetics and oral function. These findings are consistent with previous studies linking anterior tooth loss to reduced quality of life and social confidence [48, 49].

As only a few molar teeth were ultimately restored with a prosthetic replacement, it may be questioned whether the costs and resources associated with root canal treatment of these teeth were, in fact, necessary over time.

The observed patterns of prosthetic replacement following extraction have important clinical implications. As many extracted teeth, particularly molars, were not replaced, this highlights the importance of preserving tooth structure and optimising treatment strategies to maintain function. At the same time, the value of retaining molars should not be underestimated, as maintaining these teeth, even if eventually lost, may provide functional and clinical benefits over time. These findings reflect real-world clinical decision-making, where patient preferences, functional needs, and economic factors may influence whether replacement is undertaken.

This prospective cohort study, conducted within several public general dental clinics across a geographically and socioeconomically diverse Swedish region, offers several methodological strengths. First, the real-world clinical setting enhances external validity and ensures that findings reflect routine dental practice. Second, the design minimised burden on staff with limited research experience, enabling sufficient enrolment for exploratory analyses. Third, the five-year register-based follow-up allows for meaningful longitudinal analysis of prosthetic replacement patterns. Fourth, the study captured detailed baseline clinical data, including endodontic status, pain intensity, and extraction rationale, which supports nuanced investigation of factors associated with tooth loss. Fifth, the integration of digital dental records and radiographic verification strengthens data accuracy and reduces information bias.

However, certain limitations constrain the interpretability of the findings. The absence of standardised diagnostic criteria for reasons for extraction introduces variability in clinical judgement, potentially affecting internal validity. Furthermore, although electronic dental records were available, they did not capture the underlying rationale for extraction. Consequently, this study reflects recorded reasons for extraction rather than true clinical indications. Future studies with more detailed and standardised clinical documentation are needed to better assess the indications for extraction.

Additionally, the study lacked a formal sample size or power calculation, limiting its capacity to detect statistically significant subgroup differences. While the pragmatic recruitment approach supports feasibility, the resulting low proportion of replaced teeth (26%) limits the statistical robustness of analyses related to prosthetic outcomes.

These findings highlight the need for further research to better understand the clinical decision-making underlying tooth extraction, including both clinician and patient factors.

The high proportion of extracted teeth with a history of endodontic interventions warrants particular attention. Future studies should aim to explore the underlying factors contributing to this pattern in greater depth, including clinical decision-making, patient preferences, and socio-economic determinants. Such insights will be essential to inform strategies for improving tooth preservation and reduce unnecessary tooth loss.

## Conclusion

Endodontically treated teeth, encompassing both initiated treatments and prior root-filled, are overrepresented among extracted teeth in public general dental practice, although prosthetic replacement remained less common. Younger patients opted less frequently for replacement, highlighting the need for further research into decision-making processes and preferred types of prosthetic replacement.

## Supplementary information

STROBE checklist

## Data Availability

The data that support the findings of this study are available from the corresponding author, EW, upon reasonable request.
